# Hybrid Alumina–Silica Filler for Thermally Conductive Epoxidized Natural Rubber

**DOI:** 10.3390/polym16233362

**Published:** 2024-11-29

**Authors:** Hassarutai Yangthong, Phattarawadee Nun-Anan, Apinya Krainoi, Boonphop Chaisrikhwun, Seppo Karrila, Suphatchakorn Limhengha

**Affiliations:** 1 Specialized Center of Rubber and Polymer Materials in Agriculture and Industry (RPM), Department of Materials Science, Faculty of Science, Kasetsart University, Chatuchak, Bangkok 10900, Thailand; fscihty@ku.ac.th; 2 Faculty of Science and Industrial Technology, Prince of Songkla University, Surat Thani Campus, Surat Thani 84000, Thailand; phattarawadee.anan@gmail.com (P.N.-A.); apinya_may2014@outlook.com (A.K.); boonphop.cs@gmail.com (B.C.); seppo.karrila@gmail.com (S.K.)

**Keywords:** thermal conductivity, natural rubber, alumina (Al_2_O_3_), silica, hybrid filler

## Abstract

Thermally conductive composites were prepared based on epoxidized natural rubber (ENR) filled with alumina, silica, and hybrid alumina and silica. The thermal conductivity and mechanical properties were assessed. It was observed that the interactions of polar functional groups in the fillers and epoxy group in ENR supported a fine dispersion of filler in the ENR matrix. The mechanical properties were improved with alumina, silica, and hybrid alumina/silica loadings. The ENR/Silica composite at 50 phr of silica provided the highest 60 shore A hardness, a maximum 100% modulus up to 0.37 MPa, and the highest tensile strength of 27.3 MPa, while ENR/Alumina with 50 phr alumina gave the best thermal conductivity. The hybrid alumina/silica filler at 25/25 phr significantly improved the mechanical properties and thermal conductivity in an ENR composite. That is, the thermal conductivity of the ENR/Hybrid filler was 2.23 W/mK, much higher than that of gum ENR (1.16 W/mK). The experimental results were further analyzed using ANOVA and it was found that the ENR/Hybrid filler showed significant increases in mechanical and thermal properties compared to gum ENR. Moreover, silica in the hybrid composites contributed to higher strength when compared to both gum ENR and ENR/Alumina composites. The hybrid filler system also favors process ability with energy savings. As a result, ENR filled with hybrid alumina/silica is an alternative thermally conductive elastomeric material to expensive silicone rubber, and it could have commercial applications in the fabrication of electronic devices, solar energy conversion, rechargeable batteries, and sensors.

## 1. Introduction

The demand for flexible conductive materials has motivated many studies on conductive polymer-based materials. The thermal conductivity of a material is given in W/mK (Watts per meter-Kelvin). Highly thermally conductive materials are needed to make heat sinks, while materials that transfer heat slowly act as insulators. For a variety of uses, including energy conversion, heat exchange, thermal insulation, and the thermal management of electronic and photonic devices, materials with ultrahigh and ultralow thermal conductivity are preferred. The majority of polymers have relatively low thermal conductivity, which could cause major issues in device applications including heat dissipation failure, particularly for organic electronic devices. One type of polymer that is of interest for combining thermal conductivity with flexibility is natural rubber (NR). Rubber is a raw material that possesses exclusive properties and has many industrial applications. Both natural and synthetic rubbers still need research to increase their production and to improve their quality, in order to match commercial demand. Moreover, when compared to other materials (in particular, metals), these alternatives combine light weight with good process ability. However, some properties of rubber materials need improvements in order to expand their applications. This especially applies to thermal conductivity [1,2] because of its importance in many functions, such as the protective covering of electronics and sensors [1], asphalt [2], dental gloves, and foodstuff conveyor belts [3]. Rubber composites prepared with fillers have been investigated in recent studies [4,5,6], demonstrating improved thermal conductivity upon using synthetic rubber (silicone) as the matrix, chosen because silicone rubber has an excellent high-temperature resistance due to its unique molecular structure with Si–O bonds [7]. Moreover, fillers have been added in order to increase the thermal conductance of silicone products. It was found that silicone rubber showed good thermal conductivity with hybrid alumina (Al_2_O_3_) powder as a filler [8,9]. Although silicone has the advantage of high thermal conductivity, it also has disadvantages. Namely, the effects of silicone on the environment are complex and include factors related to its manufacture, use, and disposal. The use of petroleum-derived hydrocarbons in the manufacture of silicone raises carbon emissions and has an effect on sustainability. In a case study on the degradation of room-temperature vulcanizable silicone elastomers, it was found that condensation catalysts play a key role at their end-of-life stage, in both chemical and biological degradation. These compounds have a significant effect on microbial communities [10]. In addition, often, silicone rubber is too costly, having a higher price than other rubbers [11]. Therefore, it is warranted to study other rubber composite candidates for thermally conductive elastomeric materials.

Epoxidized natural rubber (ENR) as a polymer matrix has attracted attention due to its good thermal resistance [4,12] and mechanical properties similar to natural rubber [13]. ENR is produced from natural rubber by reacting it with peroxycarboxylic acid to attach epoxide groups to the isoprene units of natural rubber [14]. The epoxidation percentage of ENR can be manipulated by controlling the proportions of the reagents [15]. Nowadays, ENR is commercially produced, especially with 25% and 50% epoxidation levels. Many ENR/filler composites have been found to demonstrate improved properties, such as ENR/CB [16,17,18,19], ENR/Silica [20,21,22,23,24], ENR/carbon nanotubes (CNTs) [25,26,27], ENR/rice husk ash [28,29,30], ENR/fiber [5], and ENR/geopolymer filler composites [31]. Moreover, ENR was also used as a matrix with ceramic fillers [4,32], that is, ENR/barium titanate (BaTiO_3_) and lead titanate (PbTiO_3_) were prepared. It was discovered that the mechanical properties of the gum ENR vulcanizates and ENR/BaTiO_3_ composites were higher than those of the gum NR vulcanizate and PbTiO_3_/NR composites [33]. However, the range of applications for ENR is significantly limited by its low heat conductivity. Incorporating inorganic thermally conductive ceramic filler into polymeric composites is an effective way to increase their thermal conductivity while preserving their insulating qualities. Various hybrid fillers have been evaluated in numerous studies to enhance the thermal characteristics of ENR composites, such as CNTs–titanium isopropoxide (TiO_2_) [34] and magnesium carbonate (MgCO_3_)–silica [3]. Thermally conductive fillers with alumina were chosen [11] to address the thermal management issues.

Aluminum oxide (Al_2_O_3_) or alumina has a variety of intriguing physical and chemical qualities, such as chemical inertness, suitable hardness, good thermal and mechanical capabilities, and a reasonable price [35,36]. Recently, Al_2_O_3_ was applied in styrene–butadiene rubber (SBR) composites [37] and in silicone rubber composites [4,38,39]. The outcomes show that the thermal conductivity of silicone rubber composite increased with the loading of Al_2_O_3_ powder [38]. Nevertheless, there is poor compatibility of Al_2_O_3_ with the organic matrix, making uniform filler dispersion challenging. This affected the mechanical properties of the composites and also caused other shortcomings [40]. Therefore, the use of inorganic fillers is limited by compatibility, and improved compatibility has been sought by modifying the filler surfaces [41], by choosing the appropriate inorganic matrix [42], or by using hybrid fillers. Improved thermal conductivity and mechanical properties of the composite have been demonstrated [38].

In this present study, we propose the novel combination of an ENR matrix with alumina and silica as thermally conductive fillers. In the first case, alumina alone or silica alone is used as a filler, and in the second case, alumina and silica are used as a blend, i.e., a hybrid filler. To the best of the authors’ knowledge, this is the first instance in which a combination or hybrid filler of alumina and silica has been tested in an ENR matrix. The use of a silicone matrix or more expensive thermally conductive fillers was avoided in this study. The structure–property relationships were explored. The cure characteristics, structural and dispersion measurements (i.e., MDR, FTIR, and SEM), mechanical test results (i.e., tensile strength, 100% moduli, elongation at break, and hardness), and thermal conductivity were the evaluated properties of the ENR composites.

## 2. Materials and Methods

### 2.1. Materials

Epoxidized natural rubber with 50 mol% epoxide (ENR-50) used as the rubber matrix was manufactured by Muang Mai Guthrie Public (Surat Thani, Thailand). Aluminum oxide (Al_2_O_3_) or alumina (density = 3.94 g/cm^3^) and silica (density = 1.36 g/cm^3^), applied as fillers, were obtained from Sigma-Aldrich Chemical Co., Ltd. (Burlington, VT, USA) and from Evonik Industries (Essen, Germany). The particle sizes of ceramic materials or Al_2_O_3_ used in this work ranged from 0.4 µm to 2.4 µm. Bis(triethoxysilylpropyl)tetrasulfide, or TESPT (density of 1.09 g/cm^3^), was used as a silane coupling agent, and it was produced by Sigma-Aldrich Co. Ltd. (Dorset, UK). Butylated Hydroxyl Toluene, or BHT (density = 1.05 g/cm^3^), was used as an antioxidant and it was made by Sigma-Aldrich Chemical Co., Ltd. (St. Louis, MO, USA). Zinc oxide (ZnO) (density = 5.61 g/cm^3^) and stearic acid (density = 0.85 g/cm^3^) were utilized as activators, and were obtained from Global Chemical Co., Ltd. (Samut Prakarn, Thailand) and Imperial Industry Chemical Co., Ltd. (Pathum Thani, Thailand). Accelerators, including *N*-Cyclohexyl Benzothaizole-2-Sulfenamide or CBS (density = 1.34 g/cm^3^) and tetramethylthiuram disulfide or TMTD (density = 1.39 g/cm^3^), were manufactured by Xiamen AmoyChem Co., Ltd. (Xiamen, China) and TCI (Shanghai) Development Co., Ltd. (Shanghai, China). Sulfur (density = 2.07 g/cm^3^) was used as a curing agent. It was manufactured by Ajax Chemical (Samutprakarn, Thailand).

### 2.2. Preparation of Epoxidized Natural Rubber Composites

**Figure 1:**
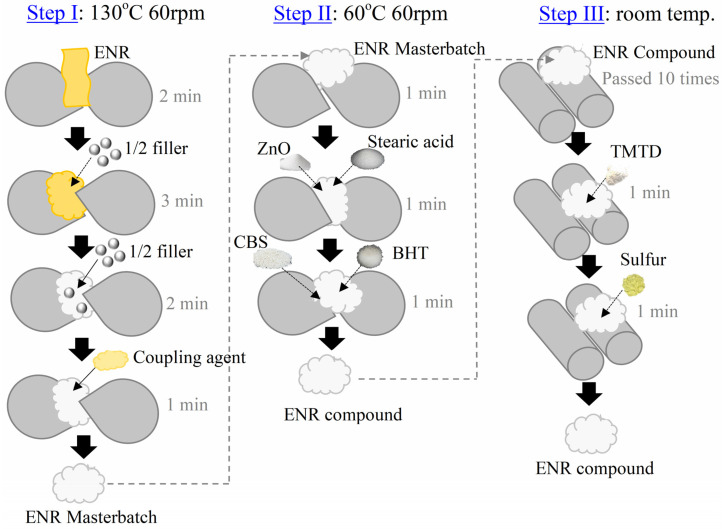
Mixing steps of ENR composites with alumina, silica, and hybrid fillers, following the formulations in Table 1.

Filled epoxidized natural rubbers (with the alternative fillers alumina, silica, and hybrid alumina/silica filler) were prepared with the formulations shown in Table 1. An internal mixer (Brabender GmbH & Co. KG, Duisburg, Germany) with a fill factor of 0.7 was used to mix ENR-50 with the filler and other chemicals at a rotor speed of 60 rpm, and the mixing steps are shown in Figure 1. First, ENR-50 was masticated at 130 °C, followed by adding filler and a coupling agent. It is noted that the filler loading was added in two parts, each providing half of the total content. After that, the masterbatch of ENR was dumped at room temperature. In the second step, masterbatch ENR was placed into the mixing chamber at 60 °C and ZnO and stearic acid were added. Then, CBS and BHT were added, and the blend was removed from the mixing chamber and allowed to cool to room temperature. In the third step, the filler dispersion was improved by passing the ENR compound ten times through the nip of a two-roll mill (Yong Fong machinery Co, Ltd., Samutsakorn, Thailand) while also mixing in TMTD and sulfur. For unfilled ENR, similar mixing was performed without filler. It is noted that the mixing time of unfilled and filled cases was controlled to 13 min. Finally, ENR composite sheets were prepared at 150 °C with compression molding (PR1D-W400L450PM, Charon Tut Co., LTD., Bangkok, Thailand). The cure time from a moving die rheometer (MDR) was used to compress each sample compound. It is noted that the samples were labeled “ENR/Alumina, ENR/Silica, and ENR/Hybrid filler”. In addition, the sample without filler was labeled “Unfilled ENR”.

### 2.3. Testing and Characterization

#### 2.3.1. ATR-FTIR Spectroscopy

The functional groups and chemical characteristics of ENR composites with alumina, silica, and hybrid fillers were evaluated utilizing Attenuated Total Reflection Fourier Transform Infrared spectroscopy (ATR-FTIR) (FTIR, Vertex70, Bruker, Karlsruhe, Germany) in transmittance mode. The spectra were taken over the range of 4000–400 cm^−1^, operated with a 4 cm^−1^ resolution and with 32 scans for each recording. The chemical alterations in the ENR composites were assessed by analyzing these spectra.

#### 2.3.2. Mooney Viscosity

Mooney viscosity measurements of ENR compounds were conducted according to the procedures described in ASTM-D 1646-89 [43]. In this work, the samples were tested by using an MV-2020 Mooney viscometer (Montech, Tokyo, Japan). The testing was performed using a large rotor at 130 °C. The sample was preheated at the test temperature for one minute before the rotor was started, and then, the Mooney viscosity (ML1+4) was recorded as the torque after the rotor had rotated for 4 min at 2 rpm (average shear rate of about 1.6 s^−1^).

#### 2.3.3. Curing Properties

The cure characteristics of the ENR composites were tested with a moving die rheometer (MDR) (Monsanto Co., Ltd., Greenville, OH, USA), according to ASTM D5289, at 150 °C, with a measurement time of 20 min. The optimum cure time (t_c_90), the scorch time (t_s_2), the minimum torque (M_L_), and the maximum torque (M_H_) are reported. The ENR vulcanizates were prepared by compression molding with a PR1D-W400L450PM compression molding machine (Charon Tut Co., LTD., Bangkok, Thailand) at 150 °C, with a compression pressure of 150 kg/cm^2^ and a time following the cure time. Finally, the ENR composite samples were conditioned in an ambient atmosphere for at least 24 h before further testing.

#### 2.3.4. Morphological Characterization

The morphological characteristics were investigated as regards the sample surface topography and composition of the composites. Morphology evaluation is one of fastest methods available for the qualitative analysis of material composition and the level of homogeneity in the sample at a given scale. The dispersion states of alumina, silica, and hybrid fillers in ENR matrix were estimated by using a field emission scanning electron microscope (FEI-Czech Republic, Oxford, UK). The ENR composites were first cryogenically cracked in liquid nitrogen. The fresh fracture surfaces were then sputter-coated with a thin layer of gold for imaging to study the surface morphology and filler dispersion.

The elemental mapping was investigated to verify the distribution of hybrid filler in the ENR matrix by using an energy-dispersive X-ray fluorescence spectrometer (Oxford Instruments, Oxfordshire, UK), combined with FE-SEM apparatus. The samples were cryogenically cracked for the study of filler dispersion, but not coated with gold.

#### 2.3.5. Mechanical Characterization

The mechanical properties (i.e., the tensile strength, modulus at 100%, and elongation at break) were investigated according to ISO 527. The test was performed using a 10ST tensile testing machine (Tinius Olsen, Salford, UK). The dumbbell shaped specimens were of the 5A-type. Testing was conducted with a crosshead speed of 200 mm/min. In addition, hardness was evaluated using a hardness testing machine (HT 3000, MonTech, Buchen, Germany). The test was run at room temperature in agreement with ISO 868. The scale of the indicating device was read after 15 s ± 1 s. Furthermore, the thermal aging properties of the ENR composites were estimated in terms of changes in the mechanical properties after aging at 100 °C for 48 h, according to ISO 188. The test results reported for each recipe are averages of five replicate samples.

#### 2.3.6. Dynamic Mechanical Properties

The dynamic mechanical properties (i.e., storage modulus and tan delta) of the ENR composites were analyzed using a DMA 8000 (Perkin Elmer Co., Ltd., Waltham, MA, USA). The samples were rectangular with a 40 mm length, 10 mm width, and 2 mm thickness. The runs were performed in tension mode at a 1 Hz frequency. The dynamic strain amplitude was 0.1%, with temperature ranging from −100 to 70 °C at a heating rate of 10 °C/min.

#### 2.3.7. Thermal Analysis

The thermal conductivity of the ENR composites was investigated. The investigation was performed using a thermal constant analyzer (TPS 2500S, Hot Disk Sweden, Goteborg, Sweden) with a Hot Disk 5465 sensor. Two samples were prepared per formulation, each of size 5 × 5 × 0.2 cm. The determinations used the transient plane source technique (TPS) [44].

#### 2.3.8. Statistical Data Analysis

The mechanical (i.e., hardness, modulus at 100%, tensile strength, and elongation at break) and thermal property results obtained from Section 2.3.5 and Section 2.3.7 were analyzed using ANOVA statistics in Excel, Microsoft 365 (Microsoft, Washington, DC, USA) with an alpha value of 0.05 (corresponding to 95% confidence level). The analysis of the data was performed from the repeated test data with 5 measurements per sample.

## 3. Results and Discussion

### 3.1. FTIR Spectroscopy for ENR Composites

**Figure 2:**
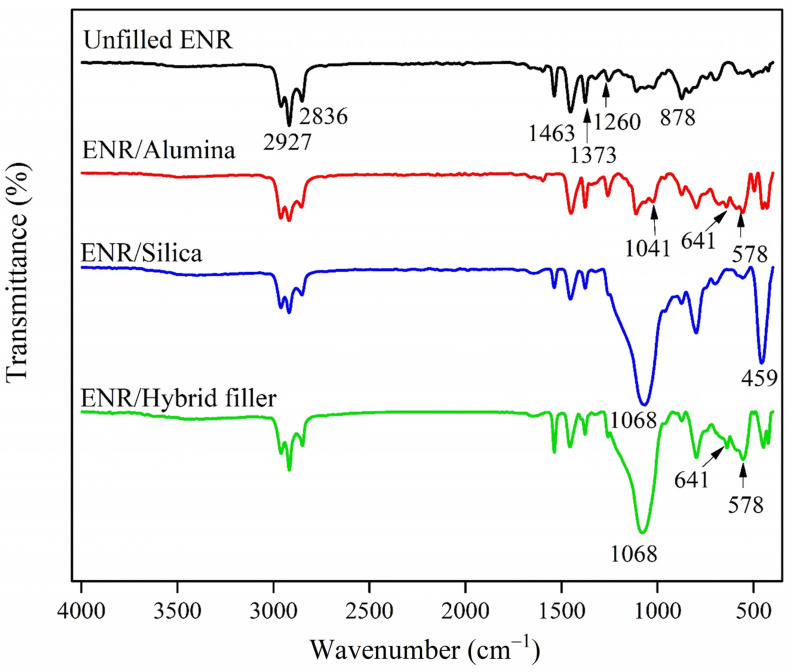
FTIR spectra of ENR composites with alumina, silica, and hybrid fillers.

**Figure 3:**
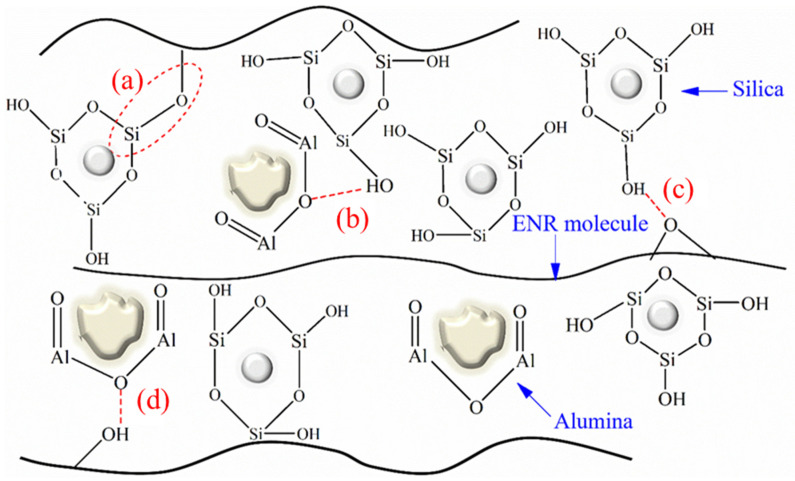
Possible chemical reactions between ENR and hybrid filler, (**a**) rubber-filler interaction, (**b**) hydrogen bonding between alumina and silica, (**c**) hydrogen bonding between ENR and silica, and (**d**) hydrogen bonding of ENR and alumina.

Figure 2 and Table 2 show the FTIR spectra of the ENR composites with alumina, silica, and hybrid fillers. The spectrum of unfilled ENR exhibited peaks of isoprene structure at 2927, 2836, and 1463 cm^−1^ (C-H stretching vibrations), and at 1373 cm^−1^ (–CH_3_ bending vibrations). The partly epoxidized rubber showed characteristic signals of epoxide units at 1260 cm^−1^ (C-O-C stretching) and 878 cm^−1^ (C-O-C ring vibration) [45]. In addition, the peaks at the wave numbers 1041, 641, and 578 for ENR/Alumina composite are characteristic of alumina, assigned to stretching modes of tetrahedral AlO_4_, whereas those near 641 cm^−1^ and 578 cm^−1^ are attributed to stretching vibrations of octahedral AlO_6_ [46,47]. For ENR/Silica composite, the relatively broad peak at 1068 cm^−1^ was for Si–O stretching vibrations of silica [48]. Moreover, the absorption peak at wavenumber 459 cm^−1^ represents amorphous silica [49], and was further investigated. The peaks in the ENR/Hybrid filler at 1068, 641, and 578 cm^−1^ were produced by the chemical properties of the ENR/Alumina/Silica mixture. In particular, the absorption peaks of the ENR/Hybrid filler composite at wavenumbers of about 1068 and 578 cm^−1^ were broader than the peaks in the spectrum of unfilled ENR. This might indicate chemical and physical interactions between the polar groups of silica and alumina particles with the oxirane groups or their opened ring products in the ENR matrix, as shown in Figure 3. In other words, during vulcanization, the silanol groups of silica interacted with the polar functional groups in epoxide rings as well as with the ring opening products of ENR [31], as shown in Figure 3a. Moreover, hydrogen bonding occurred between ENR and both fillers (Figure 3c,d), and in filler–filler interactions (Figure 3b) as well. The linkages between the ENR and functional groups of the hybrid filler were formed via rubber–filler interactions and H-bonding. The hybrid filler blend of alumina and silica had its dispersion in the ENR matrix supported by these interactions.

### 3.2. Morphological Properties

**Figure 4:**
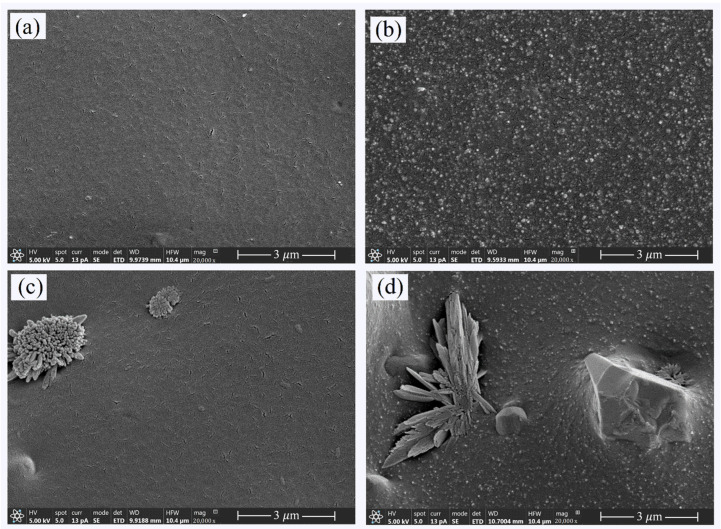
SEM micrographs of unfilled ENR (**a**) and ENR/Alumina (**b**), ENR/Silica (**c**), and ENR/Hybrid fillers (**d**) at 20,000× magnification.

The SEM micrographs of unfilled ENR, ENR/Alumina, ENR/Silica, and ENR/Hybrid filler are shown in Figure 4a–d, respectively. These show a smooth surface and some small zinc oxide particles in Figure 4a. The filler particles (i.e., silica, alumina, and hybrid fillers) are more or less uniformly distributed in the ENR matrix. In Figure 4b, it is clear that the silica-filled ENR matrix showed good filler distribution with rubber-rich areas, indicating good rubber–filler interactions (Figure 3). This is due to the polar groups of 50 mol% epoxide (ENR-50) and silanol groups of silica, as well as polar groups of alumina. Therefore, the polarity of both the filler and the rubber promoted good compatibility, as shown in Figure 4b,c. Moreover, the coupling agent was introduced to favor the homogeneous dispersion of filler in the ENR composites. This is because the dual function coupling molecules’ reactivity allows the ENR and filler to be coupled or bridged [10]. In addition, the ENR/Hybrid filler (Figure 4d) shows a fine dispersion of silica and alumina. This is attributed to the compatibility of materials and the functional groups of the coupling agent interacting with the polar groups on the hybrid filler and dominating the filler–filler interactions to prevent agglomeration. Such interactions were proven by the energy-dispersive X-ray fluorescence spectrometry.

**Figure 5:**
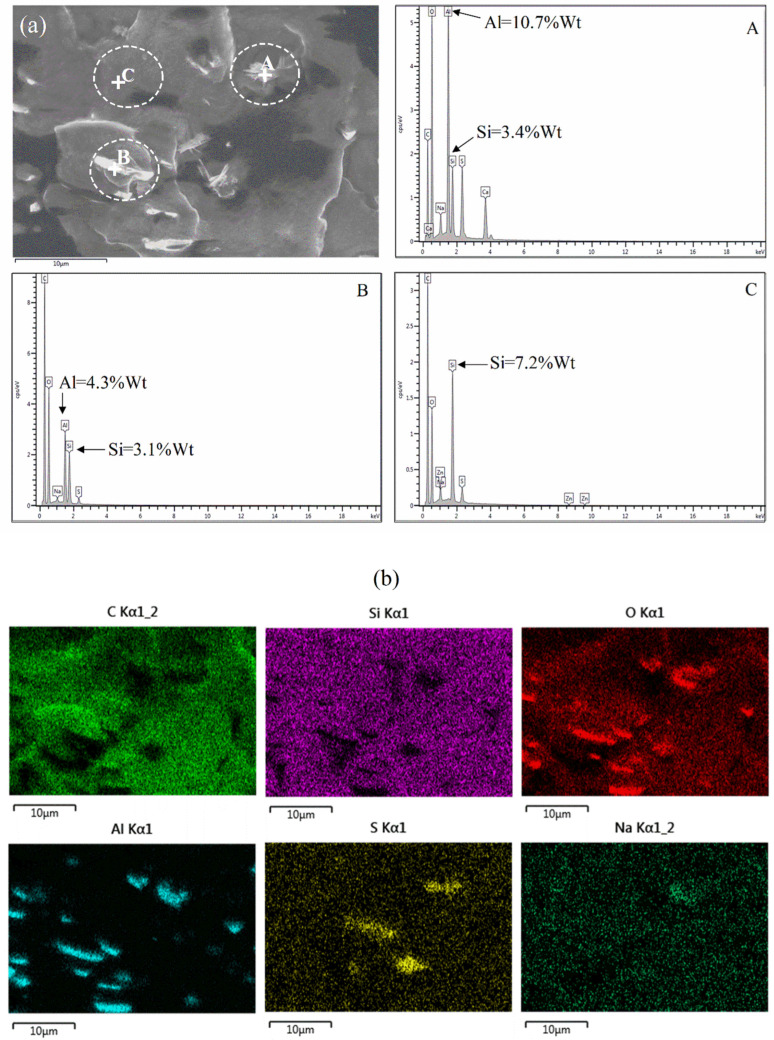
BSE-SEM (**a**) when (A)–(C) is elemental spectrum of each point on matrix, and SEM-EDX (**b**) images of ENR/Hybrid filler.

Figure 5 shows BSE-SEM and SEM-EDX images of the ENR/Hybrid filler. It was proven that the hybrid filler was comparatively uniformly distributed in the ENR matrix. In Figure 5a, the matrix of the composite has three areas selected, as shown in the BSE-SEM images. The first area (point A) shows Al and Si as the main elements in the matrix, as does point B, while the spectrum of point C shows only Si as the main element. Therefore, points A and B indicate that silica and alumina were jointly dispersed and mutually compatible. Moreover, the SEM-EDX image (Figure 5b) was further analyzed through EDX attachment to understand the miscibility in the hybrid filler–rubber matrix. That is, the elemental distributions of silica and alumina were probed by using intensity maps of Si and Al, displayed in pink and blue colors. Si was well dispersed in the ENR matrix with small particles, as was the Al element, which showed larger sized particles. This clearly presents the dispersion states of fillers in the ENR matrix. Additionally, green was used to indicate the carbon of the matrix, and yellow for the sulfur of the vulcanizing agent in the rubber recipe.

### 3.3. Cure Characteristics and Mooney Viscosity

The cure characteristics and Mooney viscosities are shown in Table 3. It was found that the compound without filler had the lowest Mooney viscosity and minimum, maximum, and delta torque, whereas the ENR composites filled with silica, alumina, and hybrid filler provided higher values. The reinforcing filler silica especially increased the torque difference. This is due to the filler (i.e., silica, alumina, or hybrid filler) increasing the stiffness and reducing the chain mobility of the ENR molecules. It should be mentioned that the crosslink density in rubber is reflected by the delta torque or torque differential [50]. In addition to crosslink density, filler–filler and filler–rubber interactions are also related to delta torque, as shown in Figure 3a–d. Therefore, increased torque suggests that there are good interactions between the filler and ENR molecules [51]. This can be mostly attributed to high compatibility between the alumina, silica, or hybrid filler and the ENR matrix, which contributed to reinforcement of the composites by the filler [52]. However, when comparing the delta of ENR/Silica and ENR/Alumina, it was found that ENR/Silica gave better delta than ENR/Alumina because of the particle size of silica. That is, silica had a larger specific surface area than alumina. Therefore, it provided more interactions with rubber molecules.

In Table 3, it is clear that the scorch times, cure times, and cure rate indexes of ENR/Alumina were close to those of unfilled ENR, and higher than those of ENR/Hybrid filler and ENR/Silica. When compared to the ENR/Silica composites, the inclusion of alumina significantly sped up the cure and scorch processes. Furthermore, it gave a very high cure rate index for the alumina-filled ENR and alumina/silica hybrid. This can be attributed to the metal oxides that, in the ENR, speed up crosslinking reactions and raise the cure rate index [53,54]. Therefore, the CRI of the ENR/Hybrid filler was between that of ENR/Silica and ENR/Alumina, with a convenient cure time. These results match the fine dispersion of both fillers, while the content of silica in the hybrid filler sample is lower than in ENR/Silica. As a result, the fillers are less likely to agglomerate.

### 3.4. Mechanical Properties

**Figure 6:**
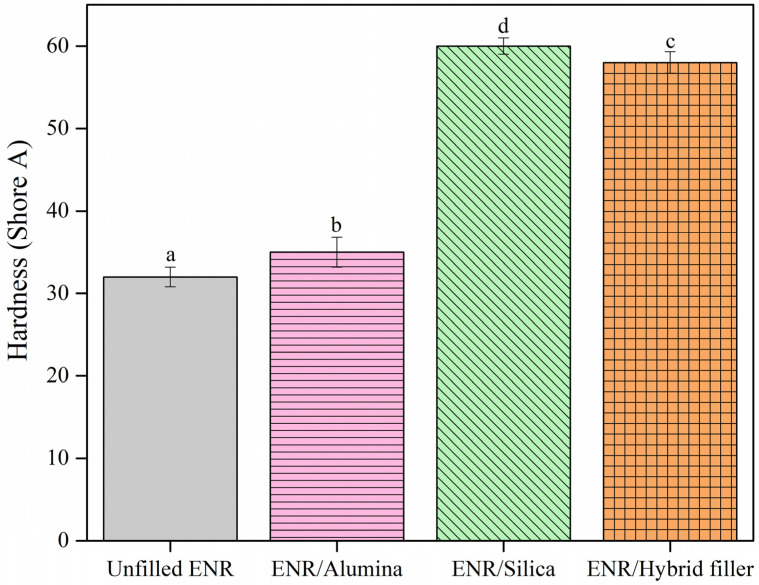
Hardness of ENR composites with alumina, silica, and hybrid fillers. a–d: Different letters within same picture indicate statistically significant differences at *p* < 0.05, based on one-way ANOVA.

**Figure 7:**
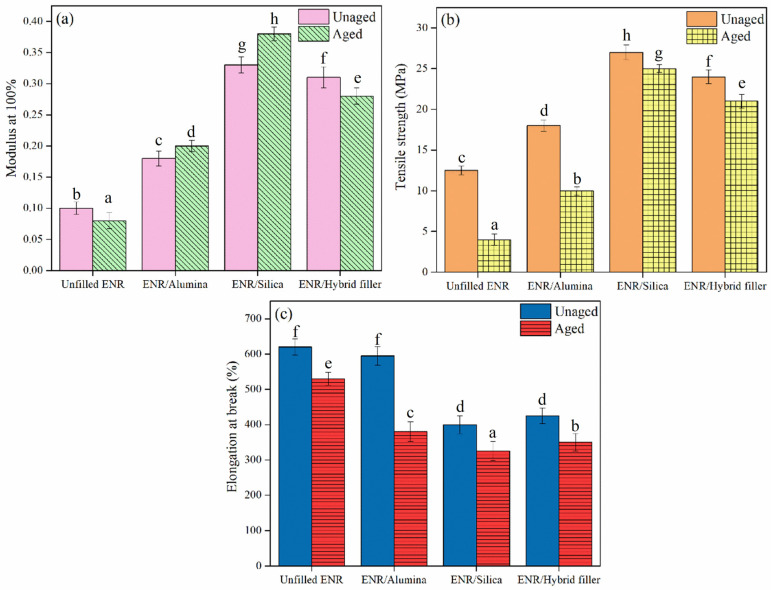
Mechanical properties: modulus at 100% (**a**), tensile strength (**b**), and elongation at break (**c**) of ENR composites, before and after aging at 100 °C for 48 h. a–h: Different letters within same picture represent statistically significant differences at *p* < 0.05, based on one-way ANOVA.

Figure 6 displays the hardness properties of ENR composites filled with alumina, silica, and hybrid fillers. It is clear that ENR/Silica composites showed the highest hardness, followed by the ENR/Hybrid filler, ENR/Alumina filler, and unfilled ENR, in that order. This shows the effects of filler particles and their interactions (silica, alumina, or hybrid filler) with the ENR matrix, as shown in Figure 3a–d. In addition, the mechanical properties, in terms of the 100% modulus, tensile strength, and elongation at break, are exhibited in Figure 7. It is seen that the 100% modulus increased with filler loading, as displayed in Figure 7a. The filler restricted the mobility of rubber chains and, hence, increased the material stiffness. Additionally, the hard filler particles in the soft matrix (the ENR matrix) were partly responsible for the rise in the modulus, whereas the interactions between the filler and rubber also contributed (Figure 3a), along with rubber–filler H-bonding (Figure 3c,d) and filler–filler interactions (Figure 3b). The silica in the ENR matrix had a smaller particle size and, accordingly, a larger effective surface area. This is consistent with the data in Table 3 showing a higher delta torque. Moreover, the tensile strength of the ENR composites showed the same trend with the 100% modulus. That is, ENR/Hybrid filler displayed lower tensile strength than the ENR/Silica composite, but higher than the ENR/Alumina composite, as seen in Figure 7b. This can be explained by the chemical interactions and hydrogen bonding between the ENR and filler, as shown in Figure 3. That is, ENR/Silica can have chemical interactions and hydrogen bonding. The presence of hydroxyl groups on the silica surfaces was detected, which can interact with the epoxide groups in ENR molecules, as shown in Figure 3a. The silanol group of silica can form an H-bond with the oxirane ring of ENR, as exhibited in Figure 3b. For ENR/Alumina, there can be H-bonding with the oxirane ring of ENR, as displayed in Figure 3d. Therefore, ENR/Hybrid filler is the most suitable formulation because a mix of silica and alumina gave no significant difference in tensile strength (i.e., 27 and 24 MPa of ENR/Silica and ENR/Hybrid filler). In addition, elongation at break, also known as the ratio of increased length to initial length at tensile failure, is depicted in Figure 7c. This shows how much bending and shaping a material can endure before breaking. It was found that the fillers decreased the maximal elongation. This may be due to the fillers decreasing the rubber content, which reduces elongation [55]. However, the filled loading in this work was controlled at 50 phr to balance the elasticity of the composites. It is seen that ENR/filler (i.e., silica, alumina, and hybrid fillers) showed lower elongation at break than unfilled ENR. This is because the filler particles reduce the chain mobility of rubber molecules and the chemical linkages between the filler items and the ENR matrix are intensified (Figure 3a), and H-bonding (Figure 3b–d) has a high reinforcing effect.

In addition, the mechanical properties after aging are among the practically important properties of rubber vulcanizates [56]. It was found that ENR/Alumina and ENR/Silica exhibited an increased 100% modulus, as shown in Figure 7a. However, tensile strength and elongation at break after aging decreased in all cases (i.e., Unfilled ENR, ENR/Alumina, ENR/Silica, and ENR/Hybrid filler), as shown in Figure 7a,b. This is due to breaking crosslinks, especially those of the polysulphidic type [57]. These are then converted to di- and mono-sulphidic bonds that are less flexible and also decrease elongation at break [58]. Moreover, the experimental results were further analyzed using ANOVA with *p* values less than 0.05 (*p* < 0.05). It was found that the hardness, 100% moduli, and tensile properties (before and after aging) of ENR/Hybrid filler exhibited significant differences. In addition, the elongation at break (before aging) of the ENR/Silica and ENR/Hybrid fillers showed no difference.

### 3.5. Thermal Properties

**Figure 8:**
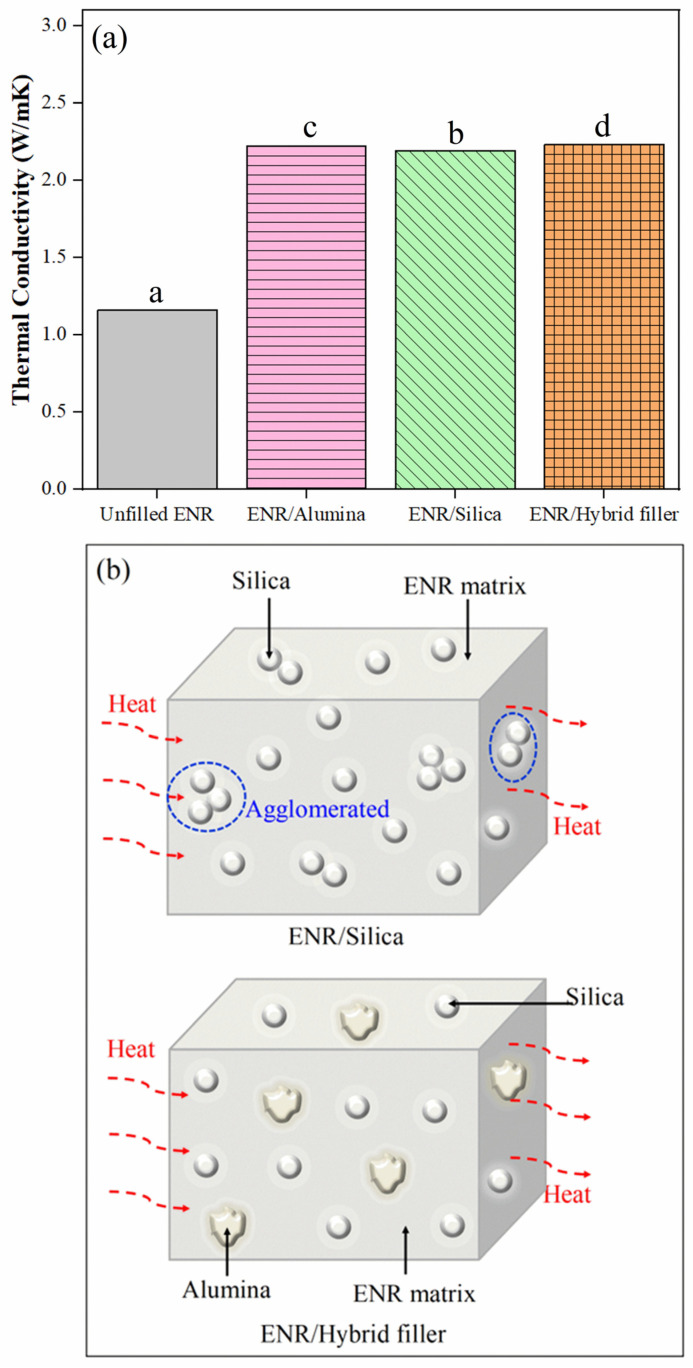
Thermal conductivity (**a**) and schematic diagram (**b**) of ENR composites. a–d: Different letters within same picture represent statistically significant differences at *p* < 0.05, based on one-way ANOVA.

Figure 8a exhibits the thermal conductivity of ENR composites with alumina, silica, and hybrid fillers. It was observed that the filler enhanced the thermal conductivity of ENR composites from that of unfilled ENR. It is clear that the thermal conductivity increased significantly on adding filler, that is, from 1.16 W/mK of unfilled ENR to 2.22, 2.19, and 2.23 W/mK of ENR/Alumina, ENR/Silica, and ENR/Hybrid filler, respectively. The ANOVA showed that all recipes conducted heat significantly differently. The filled cases had far higher thermal conductivities than the rubber matrix. This is because NR is an insulator by nature, having poor thermal conductivity. Therefore, incorporating thermally conductive particles into the ENR matrix can improve the thermal conductivity of the composite. In addition, the composites ENR/Alumina, ENR/Silica, and ENR/Hybrid filler were compared. It can be seen that ENR/Alumina showed higher thermal conductivity compared to ENR/Silica composites. This is because the thermal conductivity of silica is in the range of 0.5 to 2.5 W/mK [59], while the thermal conductivity of alumina is about 25 W/mK [60]. Moreover, ENR/Hybrid filler composites achieved the highest thermal conductivity, indicating a synergistic effect of the fillers. In addition, this might be due to the dispersion of filler, which can be explained by the schematic diagram in Figure 8b. It can be seen that the thermally conductive paths (red dashed lines) formed by fillers can pass throughout the ENR matrix. However, filler agglomeration can lower the thermal conductivity performance of the composite. Therefore, the hybrid filler that had improved dispersion in the ENR matrix gave enhanced thermal conductivity. In this work, aluminum oxide was used as a thermally conductive filler due to the polarity of the filler. It can be finely dispersed in the ENR matrix to develop a polymer composite, and with this in mind, Al_2_O_3_ was chosen because of its comparatively low cost.

### 3.6. Dynamic Properties

**Figure 9:**
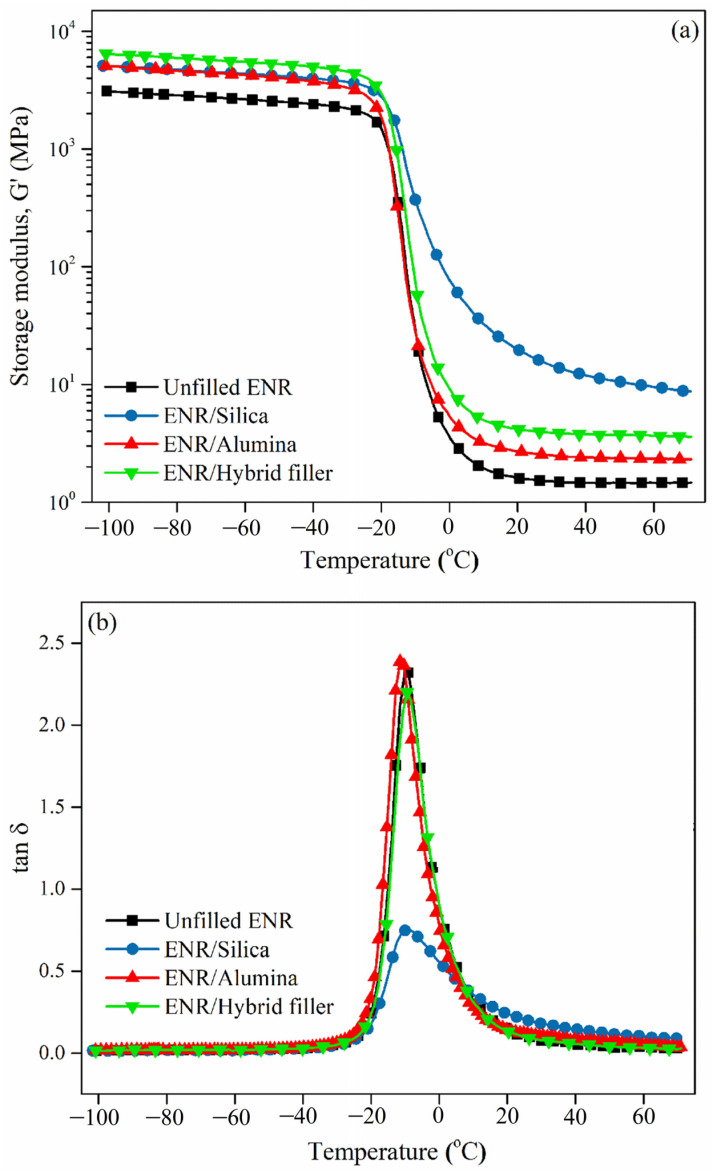
Temperature dependence of storage modulus (**a**) and of tan δ (**b**) for ENR composites with alumina, silica, and hybrid fillers.

Figure 9 shows the temperature dependences of the storage modulus (a) and tan δ (b) for the ENR composites with alumina, silica, and hybrid fillers. In general, the storage modulus of the filled rubber composite is dependent on the physical interactions between rubber molecules and inorganic filler particles [31]. In Figure 9a, the ENR/Alumina, ENR/Silica, and ENR/Hybrid fillers display higher initial storage moduli than the unfilled ENR. The storage moduli give the rank order of unfilled ENR < ENR/Alumina < ENR/Hybrid filler < ENR/Silica. This is due to the physical and chemical interactions between alumina or silica and the ENR matrix, which can be explained by the possible chemical reactions in Figure 3.

Figure 9b displays the tan δ of the ENR composites with alumina, silica, and hybrid fillers. It was found that ENR/Silica showed the lowest tan *δ*. This may be attributed to the high surface area of silica increasing the filler–rubber interactions. The decreased value of tan *δ* suggests a comparatively high filler–rubber interaction [31]. The glass transition temperature (Tg) is traditionally defined as the temperature at which the tan peak appears. It can be observed that ENR/filler (i.e., silica, alumina, and hybrid fillers) composites displayed higher *Tg* than unfilled ENR (Table 4). That is, the filler shifted the *Tg*, especially in the case of silica filler. This is due to ENR/Silica chemical interactions, partly affected by particle size, being stronger than in the ENR/Alumina case. These chemical interactions restrict the molecular mobility of rubber [61]. However, it is clear that ENR/Hybrid filler showed *Tg* values between those of ENR/Silica and ENR/Alumina. This correlates well with the mechanical properties (Figure 7).

## 4. Conclusions

In this work, a hybrid filler blend of alumina and silica was investigated for use in an epoxidized natural rubber (ENR) matrix. The formulation was compared with alumina and silica as individual fillers, as well as with unfilled ENR. The FTIR results, SEM imaging, mechanical and dynamic properties, and thermal conductivity of the ENR composites were studied. The hybrid filler showed FTIR peaks of both silica and alumina. SEM micrographs showed the dispersions of alternative fillers in the ENR matrix. SEM-EDX especially proved that silica and alumina were compatible and well dispersed. In addition, the cure characteristics displayed that the scorch time, cure times, and cure rate index of ENR/Alumina closely matched those of unfilled ENR, while adding silica (ENR/Hybrid filler) showed a CRI between ENR/Alumina and ENR/Silica. The mechanical properties hardness, 100% modulus, and tensile strength, and the DMA results for ENR/Hybrid composites, were also between those obtained with singly used fillers. Moreover, ENR/Hybrid composites exhibited higher thermal conductivity than ENR/Alumina and ENR/Silica composites. Based on ANOVA, the thermal conductivity differences were statistically significant. Therefore, the combination of alumina and silica as a hybrid filler enhanced the mechanical properties of ENR and provided enhanced thermal conductivity superior to the ENR/Silica composite, with synergistic effects from alumina and silica. This study showed that hybrid alumina and silica filler in ENR composites can be used to prepare thermally conductive materials, which normally use silicone rubber/thermal conductive filler, and this can extend the practical applications of ENR. By varying the ratio of alumina to different ceramic materials, the ENR/Hybrid Alumina/Silica composite can be used in future studies to boost thermal conductivity. Furthermore, it is interesting to investigate how matrix polarity affects a composite’s thermal conductivity by reducing the polarity of the matrix with a change from ENR-50 to ENR-25.

## Figures and Tables

**Table 1:** Compounding formulations of epoxidized natural rubber composites.

Ingredient	Quantity (phr)			
	Unfilled ENR	ENR/Alumina	ENR/Silica	ENR/Hybrid Filler
ENR 50 ^1^	100	100	100	100
Al_2_O_3_^2^	-	50	-	25
Silica	-	-	50	25
Coupling agent	-	5	5	5
Stearic acid	1	1	1	1
ZnO ^3^	3	3	3	3
BHT ^4^	1	1	1	1
CBS ^5^	1.5	1.5	1.5	1.5
TMTD ^6^	0.3	0.3	0.3	0.3
Sulfur	1.5	1.5	1.5	1.5

^1^ Epoxidized natural rubber with 50 mol% epoxide, ^2^ aluminum oxide, ^3^ zinc oxide, ^4^ Butylated Hydroxyl Toluene, ^5^ *N*-Cyclohexyl Benzothaizole-2-Sulfenamide, ^6^ Tetramethyl Thiuram Disulfide.

**Table 2:** Peak assignments in FTIR spectra for unfilled ENR, ENR/Alumina, ENR/Silica, and ENR/Hybrid fillers.

Wave Number (cm^−1^)	Assignment
2927	C-H stretching vibrations
2836	C-H stretching vibrations
1463	C-H stretching vibrations
1373	–CH_3_ bending vibrations
1260	C-O-C stretching vibrations
1068	Si–O stretching vibrations
1041	AlO_4_ tetrahedral stretching vibrations
878	C-O-C ring vibrations
641	AlO_6_ octahedral stretching vibrations
578	AlO_6_ octahedral stretching vibrations

**Table 3:** Mooney viscosity and cure characteristics of epoxidized natural rubber composites.

Sample	Mooney Viscosity (ML1+4 100 °C)	Cure Characteristics					
		M_L_^1^	M_H_^2^	M_H_-M_L_^3^	t_s2_^4^	t_c90_^5^	CRI
		(dNm)	(dNm)	(dNm)	(min)	(min)	(min^−1^)
Unfilled ENR	25.81 ± 1.31	1.05	14.24	13.19	0.30	1.23	107.5
ENR/Alumina	27.60 ± 0.92	1.92	16.63	14.71	0.29	1.11	121.95
ENR/Silica	50.20 ± 1.51	2.20	30.65	28.45	0.54	2.46	52.08
ENR/Hybrid filler	48.31 ± 1.32	2.82	27.98	25.16	0.54	2.06	65.78

^1^ Minimum torque, ^2^ maximum torque, ^3^ delta torque, ^4^ scorch time, ^5^ cure time.

**Table 4:** Maximum tan δ (tan δ_max_) and glass transition temperature (*T_g_*) for epoxidized natural rubber composites.

Sample	Tan *δ_max_* (dNm)	*T_g_* (°C)
Unfilled ENR	2.2	−10.8
ENR/Alumina	2.3	−11.2
ENR/Silica	0.8	−8.7
ENR/Hybrid filler	2.0	−9.5

## Data Availability

Data are contained within the article, further request please contact the corresponding author.
